# Telemedicine applications in pediatric emergency surgery and trauma: a systematic review of diagnostic accuracy and clinical effectiveness

**DOI:** 10.1007/s00383-025-06023-9

**Published:** 2025-04-22

**Authors:** Amani N. Alansari, Mohmed Sayed Zaazouee, Safaa Najar, Alaa Ahmed Elshanbary, Marwa Mesaoud

**Affiliations:** 1 Department of Pediatric Surgery, Hamad Medical Corporation, P.O Box: 3050, Doha, Qatar; 2 Faculty of Medicine, Al-Azhar University, Assiut, Egypt; 3 Faculty of Medicine, Alexandria University, Alexandria, Egypt; 4 Pediatric Surgery Department, Fattouma Bourguiba University Hospital, Monastir, Tunisia; 5 Faculty of Medicine of Monastir, University of Monastir, Monastir, Tunisia

**Keywords:** Telemedicine, Pediatric trauma, Emergency surgery, Systematic review, Diagnostic accuracy

## Abstract

**Aim:**

This review examines the effectiveness, implementation factors, and outcomes of telemedicine applications in managing surgical emergencies and trauma in pediatric patients.

**Methods:**

We conducted a systematic review following PRISMA guidelines. Comprehensive searches were performed across four major databases (PubMed, Scopus, Web of Science, and Cochrane Library) from inception to January 2025. Studies that investigate telemedicine interventions in pediatric (ages 0–18) emergency surgery and trauma settings were included.

**Results:**

Eleven studies met inclusion criteria, comprising 6 retrospective cohorts, 3 cross-sectional studies, 1 prospective cohort, and 1 case series. Diagnostic accuracy was consistently high across applications (sensitivity: 93.8–100%; specificity: 82.6–99.7%). Telemedicine demonstrated effectiveness in preventing unnecessary transfers, with one study reporting 16 prevented transfers. Remote-guided procedures showed comparable accuracy to in-person assessment, particularly in point-of-care ultrasound (sensitivity: 93.8%, specificity: 99.7%). High satisfaction rates were reported among families and healthcare providers.

**Conclusions:**

This review highlights the potential of telemedicine in pediatric emergency surgery and trauma care, particularly in enhancing access to specialized care and maintaining diagnostic accuracy. However, the generalizability of findings remains limited due to variations in study methodologies. Future research should emphasize prospective designs and economic evaluations to clarify the cost–benefit ratio of implementation. Establishing standardized protocols and enhancing provider training will be essential for the effective and sustainable integration of telemedicine into pediatric trauma systems.

**Supplementary Information:**

The online version contains supplementary material available at 10.1007/s00383-025-06023-9.

## Introduction

The rapid evolution of digital healthcare technologies has transformed medical practice across various specialties, with telemedicine emerging as a pivotal innovation in healthcare delivery [1, 2]. Pediatric emergency surgery and trauma care demand swift, precise decision-making and specialized expertise that may not be readily available in all healthcare settings, particularly in remote or resource-limited areas [1, 3]. Recent global events, including the healthcare access restrictions imposed by the COVID-19 pandemic, have accelerated the adoption of telemedicine solutions across medical disciplines [4–6]. However, the specific application of these technologies in pediatric surgical emergencies requires careful evaluation to ensure optimal patient outcomes.

The unique considerations in pediatric emergency care, including age-specific physiological responses, anatomical variations, and the critical role of timely intervention, make this population particularly sensitive to healthcare delivery methods [7, 8]. Telemedicine applications in this context range from remote surgical consultation and triage to postoperative monitoring and follow-up care [9, 10]. Understanding the effectiveness, limitations, and best practices of these applications is crucial for healthcare providers, administrators, and policy makers.

Previous research has demonstrated varying degrees of success in implementing telemedicine solutions across different medical specialties [11–13]. Studies have shown promising results in adult emergency care, with evidence supporting improved access to specialized care, reduced transfer times, and enhanced decision-making processes [14, 15]. However, the extrapolation of these findings to pediatric emergency surgery and trauma requires careful consideration of the unique challenges posed by this vulnerable population.

The objectives of this systematic review are threefold: first, to evaluate the effectiveness of telemedicine applications in pediatric emergency surgery and trauma care; second, to identify factors influencing the success or failure of these implementations; and third, to assess the impact of telemedicine on patient outcomes, healthcare resource utilization, and cost-effectiveness.

## Patients and methods

This systematic review was conducted following the Preferred Reporting Items for Systematic (PRISMA) guidelines and the Cochrane Handbook [16, 17].

### Eligibility criteria

Studies were included based on the Population, Intervention, Comparison, Outcomes, and Study Design (PICOS) framework. The population comprised pediatric patients (aged 0–18 years) in surgical emergency and trauma settings. The intervention involved the use of telemedicine for consultation, diagnosis, or treatment decision-making. Comparisons were made against standard in-person clinical care, historical cohorts, non-telemedicine-based consultations, or no control group. Outcomes assessed included diagnostic accuracy, clinical decision-making, time to treatment initiation, patient outcomes, and resource utilization. Eligible study designs included randomized controlled trials (RCTs), retrospective and prospective cohort studies, cross-sectional studies, and case series. Studies were excluded if they were review articles, editorial comments, non-English articles, conference abstracts, or focused on non-trauma-related conditions.

### Information sources and search strategy

A comprehensive literature search was conducted in PubMed, Scopus, Web of Science, and Cochrane Library from inception to January 2025. The search strategy was developed using a combination of Medical Subject Headings (MeSH) terms and keywords related to telemedicine, pediatric trauma, and surgical emergency care. Boolean operators (AND, OR) were applied to refine the search. Detailed search strategies are shown in supplementary Table 1.

### Study selection

All retrieved articles were imported into EndNote (Version 20), and duplicates were removed. Two independent reviewers screened the titles and abstracts for relevance. Full texts of potentially eligible studies were assessed against the inclusion and exclusion criteria. Disagreements were resolved through discussion or consultation with a third reviewer.

### Data collection process and data items

Data extraction was performed independently by two reviewers using a standardized data extraction sheet. Extracted variables included study characteristics (author, year, country), study design, sample size, patient demographics, type of trauma, telemedicine intervention details, control group characteristics, and primary outcomes. Any discrepancies were resolved through discussion.

### Risk of bias assessment

Diagnostic accuracy studies were assessed using the Quality Assessment of Diagnostic Accuracy Studies-2 (QUADAS-2) tool, which evaluates the risk of bias and applicability concerns across four key domains: patient selection, index test, reference standard, and flow and timing [18]. Cohort studies were evaluated using the Newcastle–Ottawa Scale (NOS), a tool that assesses study quality based on three criteria: selection of participants, comparability of groups, and outcome assessment [19]. Case series were assessed using the Joanna Briggs Institute (JBI) critical appraisal checklist, which examines study clarity, patient inclusion criteria, and the reliability of reported outcomes [20]. Each study was independently rated by two reviewers for methodological quality and risk of bias, with any disagreements resolved through discussion.

### Qualitative analysis

A thematic analysis approach was used to synthesize qualitative findings related to the implementation and effectiveness of telemedicine in pediatric surgical emergency and trauma settings. Recurring themes such as improved access to care, diagnostic accuracy, clinical workflow efficiency, and patient satisfaction were identified and analyzed across studies. A framework analysis method was applied to organize key insights, facilitating a deeper understanding of the contextual factors influencing telemedicine adoption in pediatric trauma care.

## Results

**Fig. 1:**
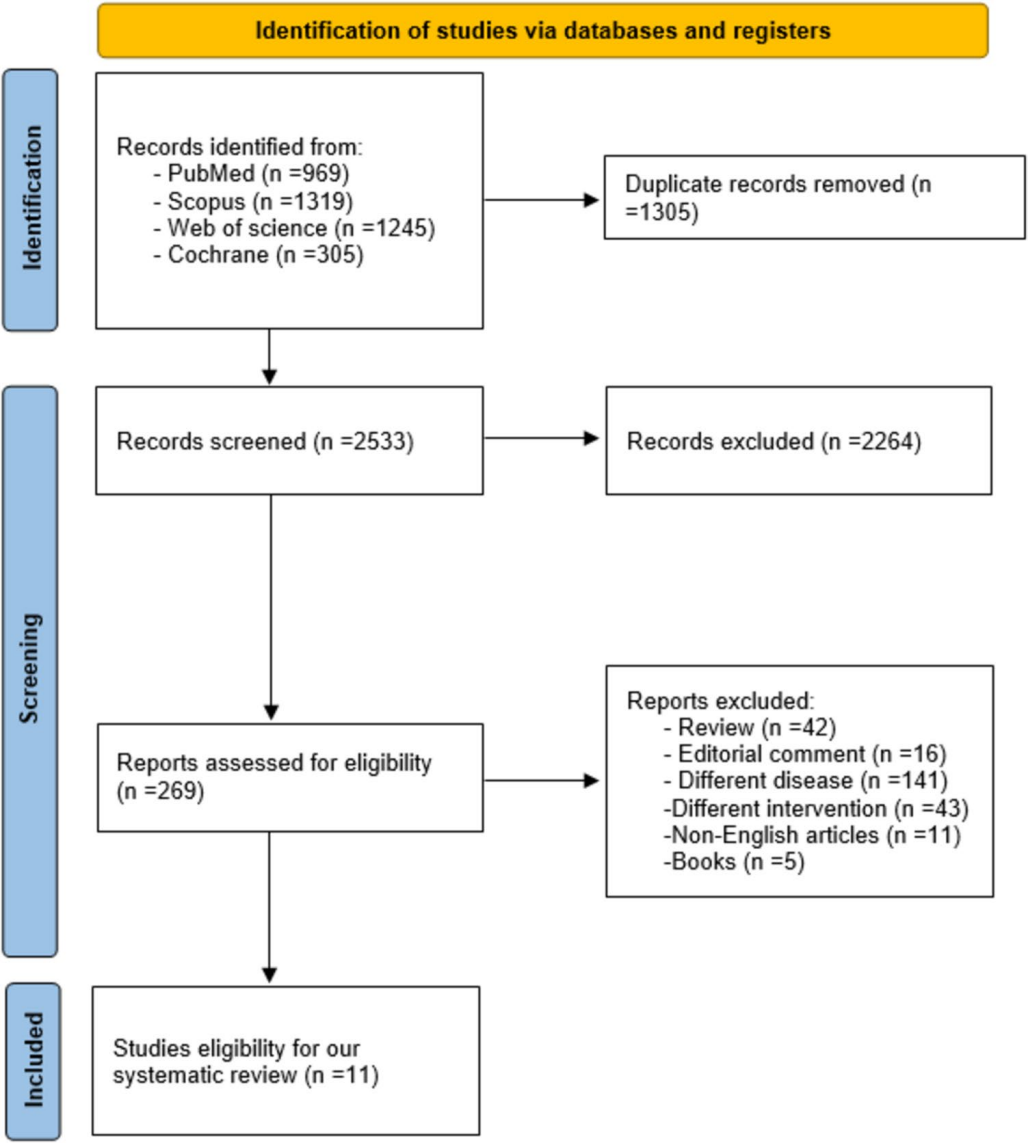
Flow diagram of the study selection process

A total of 3838 records were identified from PubMed (969), Scopus (1319), Web of Science (1245), and Cochrane (305). After removing 1,305 duplicates, 2533 records were screened by title and abstract, with 2264 excluded. Full-text assessment was conducted for 269 reports, leading to the exclusion of 258 based on criteria such as review articles (42), editorial comments (16), different diseases (141), different interventions (43), non-English articles (11), and books (5). Ultimately, 11 studies met the eligibility criteria for inclusion [11–13, 21–28]. Figure 1

### Baseline and summary of the included studies

The included studies encompass various designs, including 6 retrospective cohorts, 3 cross-sectional studies, one prospective cohort, and one case series, conducted across multiple countries, including France, the USA, the UK, Italy, and Russia. Sample sizes range from 15 to 143 pediatric patients, covering diverse conditions such as orthopedic trauma, facial lacerations, intracranial hemorrhages, and abdominal injuries. Telemedicine tools include video conferencing, mobile applications, digital imaging transmission, and tele-ultrasound. Control groups vary, including non-telemedicine care, historical cohorts, and bedside assessments. Primary outcomes focus on diagnostic accuracy, clinical decision-making, time-to-surgery, and telemedicine effectiveness. Specialties involved include orthopedics, radiology, neurosurgery, emergency medicine, and ophthalmology, with injury locations spanning the limbs, head, spine, chest, and abdomen. Table 1

**Table 1:** Summary of the included studies

Study ID	Study design	Study site	Total number of patients	Population details	Telemedicine details	Control details	Primary Outcome	Specialty admitted under	Injury location
Martino [12]	Retrospective cohort	USA	42	Pediatric trauma patients < 18 years	Tool: 1-Teleradiology servers for CT scan review 2-Radiology resident (RR) vs. attending teleradiologist (AT) interpretations 3-Electronic medical record (EMR) integration Action: 1-Overnight teleradiology support for CT imaging. 2-Comparison of RR and AT interpretations for accuracy/timing	CT scan interpretations by a radiology resident	Discrepancy rate	Radiology	Combined/multiple injuries
Zorin [28]	Retrospective cohort	Russia	78	Pediatric patients with musculoskeletal injuries requiring urgent/emergent consultations	Tool: 1-Telemedicine Consultations (TMC): Used for virtual evaluations of pediatric musculoskeletal injuries. Actions: 1-Retrospective Review: Analyzed 3,745 TMC requests (2020–2022) by urgency (572 urgent/emergent), region, and alignment with diagnoses. 2-Outcome Evaluation: Assessed TMC effectiveness in trauma care and patient routing for pediatric cases	NR	Effectiveness of TMC	Orthopedics	Combined/multiple injuries
Taylor [25]	Retrospective cohort	USA	71	Pediatric trauma patients (7 months–15 years)	Tool: 1-Mobile application/computer for consultation requests 2-Video conferencing software 3-Pediatric trauma advanced practice provider (APP) 4-Pediatric neurosurgeon collaboration Action: 1-Video conferencing for patient history, exam, and imaging review. 2-Telehealth consultations to guide treatment/disposition decisions. 3- Monthly reviews to assess telehealth impact on patient transfers	1-Children admitted without telehealth consultation (per head trauma protocol). 2-Children transferred without telehealth consultation	Prevention of unnecessary transfers	Emergency	Head, chest, skull, and abdomen
Whitney [26]	Cross-sectional	USA	130	Pediatric patients undergoing ultrasound scans for abdominal free fluid, intussusception, or hip effusions	Tool: 1-iPhone 3G devices 2-Skype 3–6-s.mp4 video clips (FAST scans, intussusception, hip effusions) 4-Pediatric emergency department database Action: 1-Real-time remote ultrasound interpretation via live-stream video feed. 2-Two-way audio for requesting pauses/replays of clips	Bedside ultrasound	Interrater reliability	Radiology	Abdomen
Jackson [11]	Retrospective cohort	USA	15	Children ≤ 18 years transferred for operative intracranial hemorrhage requiring neurosurgery within 2 h of arrival	Tool: 1-Departmental smartphones for telemedicine 2-Synchronous (real-time video/audio) and store-and-forward (image transfer) methods 3-Electronic health records (EHRs) for data analysis Action: 1-Transmitting neuroimaging and clinical updates to neurosurgical teams during interhospital transport. 2-Case-by-case decision-making on imaging sharing by pediatric transport physicians. 3-Retrospective analysis of time-to-surgery outcomes for intracranial hemorrhage patients	Non-telemedicine group	Time to surgery	Radiology	Brain
Zennaro [27]	Prospective cohort	Italy	52	Children aged 0–18 years in the pediatric ED. Inclusion: 8 clinical scenarios (e.g., traumatic abdomen, suspected appendicitis, hip pain)	Tool: 1-Tele-ultrasound (TELE POC) with low-cost commercial off-the-shelf (COTS) equipment 2-Open-source software for real-time radiologist guidance 3-Predefined templates for data collection Action: 1-Remote guidance of pediatricians during point-of-care ultrasound exams. 2-Three consecutive ultrasound exams per child (remote-guided, radiologist-led, and blind radiologist review). 3-Prospective data collection and inter-observer agreement analysis for diagnostic accuracy	Radiologist-led scans	Diagnostic accuracy	Radiology	Abdomen
Farook [22]	Retrospective cohort	UK	143	Children with facial lacerations	Tool: 1-Telemedicine photographs of facial lacerations 2-Proforma for data collection 3-SPSS for analysis Action: Comparing management outcomes with/without telemedicine photos	Verbal referrals	clinical decision-making	Oral and Maxillofacial Surgery and plastic surgery	Face
Elkaim [21]	Case series	France	20	Children with acute orthopedic trauma	Tool: 1-Mobile phone digital camera. 2-Multimedia Messaging Service (MMS). 3-Computerized database for storage. Action: 1-Transmitting X-rays via MMS for urgent orthopedic consultations. 2-Phone conference to discuss transfer needs and management plans	NR	Agreement on diagnosis/management	Orthopedics	Limbs, spine
Saleh [13]	Cross-sectional	France	21	Children < 3 years suspected of abusive head trauma	Tool: 1-RetCam 120 for fundus imaging 2-Digital image transmission for remote reading 3-Ophthalmologist comparison of RetCam vs. ophthalmoscopy findings Action: 1-Remote diagnosis of retinal hemorrhages via transmitted images. 2-Classification of fundus abnormalities (subtle/moderate/severe)	Standard ophthalmoscopy	Diagnostic accuracy	Ophthalmology	Head/brain
Marcin [23]	Retrospective cohort	USA	97	Pediatric trauma patients ≤ 16 years admitted to adult ICU	Tool: 1-Live two-way audiovisual consultations 2-Pediatric critical care physician notes 3-Trauma database (MMCR) for data analysis Action: 1-Comprehensive remote patient evaluation (history, vitals, imaging). 2-Satisfaction surveys for stakeholders (parents, providers)	Historical cohort treated without telemedicine	Effectiveness and satisfaction	Critical Care	Combined/multiple injuries
Palombo [24]	Cross-sectional	UK	30	Children < 14 years with minor trauma (no major trauma cases)	Tool: 1-Videoconferencing link 2-Document camera with backlight for radiograph transillumination 3-Standard viewing box for comparison Action: 1-Remote radiograph interpretation with clinical history. 2-Comparison of telemedicine vs. direct radiograph diagnosis	Radiographs viewed via standard viewing box	Diagnostic accuracy	Radiology	Limbs, face and skull

Not reported (NR)

The baseline characteristics of the included studies vary widely. Patient ages range from 0.39 to 13.3 years. GCS is reported in two studies, ranging from 6 to 12.8. Injury Severity Scores range from 10.4 to 31. Male predominance is common, ranging from 46.66% to 71.4%. Falls are a frequent injury mechanism, reported in up to 40.5% of cases. Table 2

**Table 2:** Baseline characteristics of the included studies

Study ID	Study groups	Age in years, mean (SD)	GCS, mean (SD)	Injury severity score, mean (SD)	Sex (male), no. (%)	Fall as a mechanism of injury, no. (%)
Martino [12]	Telemedicine group (135)	NR	NR	NR	NR	NR
	Resident radiology group (135)	NR	NR	NR	NR	NR
Zorin [28]	All (347)	NR	NR	NR	NR	NR
Taylor [25]	Telemedicine group (8)	(0.58–15) *	NR	NR	NR	NR
	No telemedicine admission group (8)	(0.58–15) *	NR	NR	NR	NR
	No telemedicine transfer group (56)	NR	NR	NR	NR	NR
Whitney [26]	Telemedicine group	NR	NR	NR	NR	NR
	No telemedicine group	NR	NR	NR	NR	NR
Jackson [11]	Telemedicine group (8)	4.01 (3.48)	8.5 (4.02)	NR	4 (50)	NR
	No telemedicine group (7)	3.33 (2.67)	6 (4.59)	NR	5 (71)	NR
Zennaro [27]	Telemedicine group (52)	8.1 (7.54)	NR	NR	30 (57.7)	NR
	No telemedicine unblinded radiologist (52)		NR	NR		NR
	No telemedicine blinded radiologists (52)		NR	NR		NR
Farook [22]	Telemedicine group (98)	6.62 (4.14)	NR	NR	68 (70)	39 (40.5)
	No telemedicine group (45)	4.5 (3.56)	NR	NR	28 (63)	19 (42)
Elkaim [21]	Cases data (20)	9.25 (3.5)	NR	NR	13 (65)	NR
			NR	NR		NR
Saleh [13]	Telemedicine group (21)	0.39 (0.47)	NR	NR	15 (71.4)	6 (28.5)
	Standard Ophthalmoscopy (21)		NR	NR		
Marcin [23]	Telemedicine group (17)	5.5 (4.1)	11.6 (3.9)	18.3 (8.3)	NR	4 (23.5)
	Historical no telemedicine group (127)	12.8 (5.3)	10.4 (3.6)	10.4 (9.1)	NR	14 (11.0)
	No telemedicine group (80)	13.3 (3.9)	12.8 (3.8)	14.7 (10.8)	NR	7 (8.8)
Palombo [24]	Cases data (30)	10 (3)	NR	NR	14 (46.66)	NR

Glasgow Coma Scale (GCS), number (no.), not reported (NR), range (*)

### Quality assessment

**Fig. 2:**
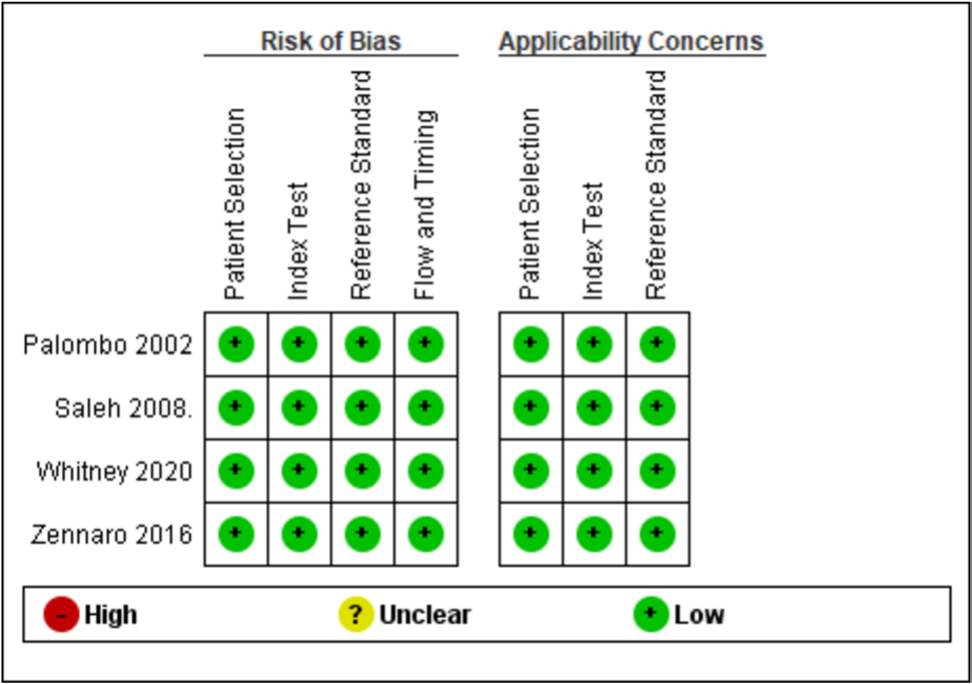
Risk of bias assessment using the ROB 2 tool

The four included diagnostic studies were assessed using the QUADAS-2 tool and were judged to have a low risk of bias across all domains (Fig. 2). Among the retrospective cohort studies, evaluated using the Newcastle–Ottawa Scale (NOS), five were rated as having good quality, while Jackson et al.’s [11] study was classified as poor quality (Table 3). The case series by Elkaim et al. [21], assessed using the Joanna Briggs Institute (JBI) critical appraisal checklist, was determined to be of moderate quality (Table 4).

**Table 3:** Quality assessment of the included cohort studies using the Newcastle–Ottawa scale

Study ID	Selection				Comparability	Outcome			Quality score
	D1	D2	D3	D4		D5	D6	D7	
Martino [12]	*	*	*	*	*	*	*	*	Good
Zorin [28]	*	*	*	*	*	*	*	*	Good
Taylor [25]	*	*	*	*	*	*	*	*	Good
Jackson [11]	*	*	*	*		*	*	*	Poor
Farook [22]	*	*	*	*	**		*	*	Good
Marcin [23]	*	*	*	*	*	*	*	*	Good

D1: Is the case definition adequate/representative of the exposed cohort?

D2: Representative of the cases/selection of the non-exposed cohort

D3: Selection of controls/ascertainment of exposure

D4: Definition of controls/ demonstration that outcome of interest was not present at the start of the study

D5: Ascertainment of exposure/assessment of outcome

D6: Same method of ascertainment for cases and controls/Was follow-up long enough for outcomes to occur?

D7: Non-response rate/adequacy of follow-up of cohorts

**Table 4:** Quality assessment of case series study using the Joanna Briggs Institute checklist

Study ID	D1	D2	D3	D4	D5	D6	D7	D8	D9	D10	Overall quality assessment
Elkaim [21]	Yes	Yes	Yes	No	No	Yes	Yes	No	Yes	No	Moderate

D1: Were there clear criteria for inclusion in the case series?

D2: Was the condition measured in a standard, reliable way for all participants included in the case series?

D3: Were valid methods used for identification of the condition for all participants included in the case series?

D4: Did the case series have consecutive inclusion of participants?

D5: Did the case series have complete inclusion of participants?

D6: Was there clear reporting of the demographics of the participants in the study?

D7: Was there clear reporting of clinical information of the participants?

D8: Were the outcomes or follow-up results of cases clearly reported?

D9: Was there clear reporting of the presenting site(s)/clinic(s) demographic information?

D10: Was statistical analysis appropriate?

### Qualitative synthesis

#### Improved access to care

Telemedicine has significantly enhanced access to emergency surgical care for pediatric patients, particularly in rural and underserved areas. Zorin et al. [28] reported a threefold increase in telemedicine consultation requests over 3 years, indicating a growing reliance on this technology to connect patients with specialized care. The study highlighted that 3745 telemedicine requests were received, with 572 categorized as urgent or emergent. This increase reflects the potential of telemedicine to bridge the gap in healthcare access, allowing timely interventions that can prevent unnecessary transfers to distant trauma centers [28]. Similarly, Taylor et al. [25] noted that their telemedicine program prevented 16 unnecessary transfers, demonstrating how telemedicine can keep patients closer to home while still receiving appropriate care [25]. Furthermore, Elkaim et al. [21] emphasized that teleconsultation using Multimedia Messaging Service (MMS) is particularly beneficial for improving remote management of orthopedic patients, allowing for timely decisions regarding transfers and treatment plans [21]. Palombo et al. [24] highlighted the role of telemedicine in facilitating pediatric radiograph consultations, particularly in emergency settings where immediate specialist access is crucial [24]. Martino et al. [12] highlighted the role of telemedicine in providing timely interpretations of pediatric trauma CT scans, which is crucial for effective management and treatment decisions. Their study found that the mean time to interpretation (TTI) was significantly shorter for radiology residents (55.9 min) compared to attending teleradiologists (90.4 min, *P* < 0.001).

#### Diagnostic accuracy and reliability

The accuracy of remote consultations in pediatric trauma has been a focal point in recent studies. Zennaro et al. [27] found that point-of-care ultrasound (POC US) performed by pediatricians under remote guidance from radiologists yielded a sensitivity of 93.8% and specificity of 99.7% when compared to expert radiologists [27]. This high diagnostic accuracy highlights telemedicine's effectiveness in providing timely and precise assessments, crucial in emergency settings where every minute counts. Whitney et al. [26] reported strong interrater reliability for ultrasound interpretations between bedside and remote evaluators across various pediatric conditions. The κ values for focused assessment with sonography in trauma, intussusception, and hip effusion were 0.748, 0.816, and 0.764, respectively [26]. Percent agreement adjusted for chance was 86%, 80%, and 88%, with κ values of 0.851, 0.8, and 0.747, respectively. Further adjustments revealed agreements of 92%, 81%, and 88%. Additionally, Saleh et al. [13] demonstrated that digital camera imaging of the eye fundus for suspected abusive head injuries achieved a sensitivity of 100% and specificity of 85.7%, further supporting the reliability of telemedicine in pediatric emergency care [13]. Palombo et al. [24] found that telemedicine for pediatric radiographs had a sensitivity of 98.6% and specificity of 82.6%, indicating that telemedicine can effectively aid in diagnosing pediatric injuries [24]. Martino et al. [12] found that radiology residents had comparable discrepancy rates in interpreting pediatric trauma CT scans to attending teleradiologists, demonstrating that telemedicine can maintain diagnostic accuracy across different levels of expertise. Discrepancy (13.3% vs. 13.3%), major discrepancy (4.4% vs. 4.4%), missed findings (9.6% vs. 12.6%), and overcalls (3.7% vs. 0.7%) were similar between residents and attending teleradiologists, with no statistically significant differences (all *P* > 0.05) [12].

#### Enhanced training and education

Telemedicine serves as a valuable educational tool for healthcare providers. In the study by Taylor et al. [25], the implementation of a pediatric trauma telemedicine program led to increased comfort among emergency medicine providers in managing head-injured patients [25]. The program included regular educational sessions, which helped improve the knowledge and skills of the staff at the partnering hospital. This aspect of telemedicine is vital, as it promotes a collaborative learning environment where providers can enhance their competencies while delivering care. Similarly, Marcin et al. [23] highlighted the importance of telemedicine in providing pediatric critical care consultations, emphasizing that telemedicine can help bridge the knowledge gap for providers in rural settings, ultimately improving patient outcomes [23]. Palombo et al. [24] also noted that the use of telemedicine in interpreting pediatric radiographs can serve as a training opportunity for emergency department staff, enhancing their diagnostic skills [24]. Martino et al. [12] suggested that focused training for radiology residents in pediatric trauma imaging is essential to improve interpretation accuracy, indicating the need for ongoing education in telemedicine applications [12].

#### Cost-effectiveness and resource utilization

Jackson et al. [11] highlighted that telemedicine in interhospital transport accelerates definitive care for pediatric intracranial hemorrhage, potentially improving outcomes, reducing hospital stays, and enhancing cost efficiency [11]. Palombo et al. [24] also suggested that the use of telemedicine could reduce the need for unnecessary transfers and consultations, ultimately saving costs for healthcare systems [24].

#### Patient and family satisfaction

Farook et al. [22] reported high levels of satisfaction among families who utilized telemedicine for pediatric facial lacerations, with 86% (Vs 82% in the verbal consultation group) of patients undergoing surgery after teleconsultation. The ability to consult with specialists without the need for travel can reduce the stress and anxiety associated with emergency care, allowing families to remain close to their support systems during critical times [22]. This aspect of telemedicine is particularly important in pediatric care, where the emotional well-being of both the child and family is important. Moreover, the study by Marcin et al. [23] indicated that telemedicine consultations resulted in high satisfaction rates among parents and providers, reinforcing the positive impact of telemedicine on the overall healthcare experience [23]. Palombo et al. [24] also found that the use of telemedicine for pediatric radiographs led to high satisfaction levels among emergency department staff, as it facilitated quicker consultations and improved patient management [24].

#### Challenges and limitations

**Fig. 3:**
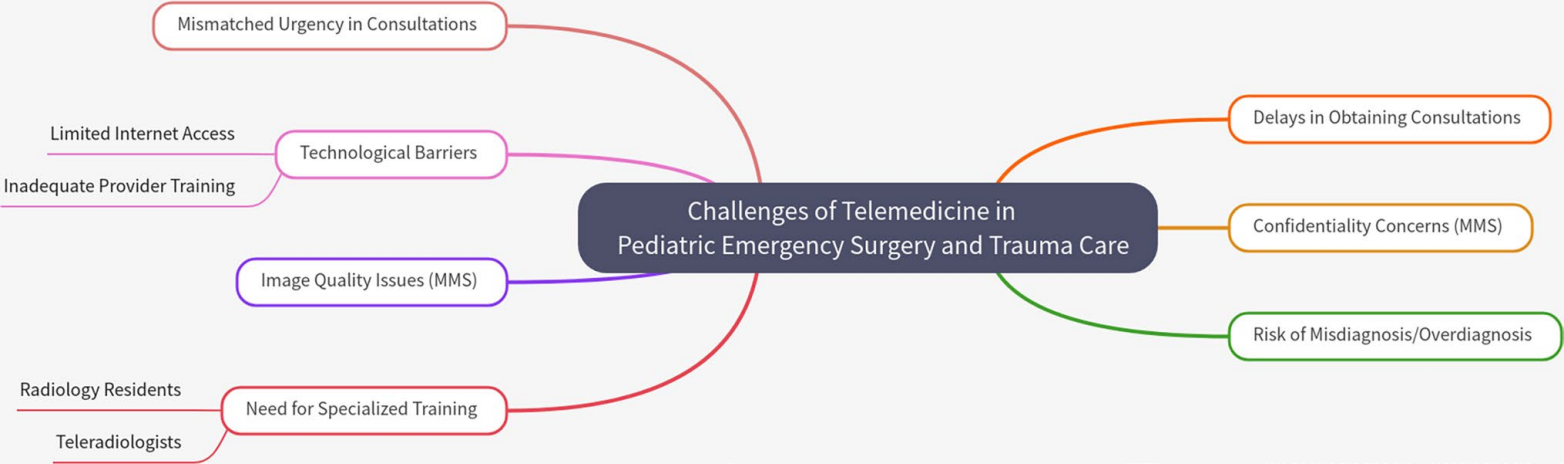
Challenges and limitations of pediatric surgical emergency and trauma care

Despite the numerous benefits, challenges remain in the implementation of telemedicine in pediatric trauma care. Zorin et al. [28] identified issues such as mismatched urgency categories in consultation requests and delays in obtaining telemedicine consultations. These challenges can hinder the effectiveness of telemedicine, potentially leading to adverse outcomes for patients. Additionally, the reliance on technology can pose barriers in areas with limited internet access or inadequate training for healthcare providers [28]. Elkaim et al. [21] also noted that while teleconsultation using Multimedia Messaging Service (MMS) was effective, concerns about patient confidentiality and the quality of transmitted images remained significant challenges [21]. Palombo et al. [24] highlighted that while telemedicine can aid in diagnosing pediatric injuries, there is still a risk of misdiagnosis or overdiagnosis, which can lead to unnecessary treatments [24]. Martino et al. [12] further emphasized that both radiology residents and attending teleradiologists require focused training in pediatric trauma imaging to mitigate discrepancies in interpretations [12] (Fig. 3).

#### Integration of technology and training

Zennaro et al. [27] emphasized the need to train pediatricians in point-of-care ultrasound under remote guidance to ensure accurate diagnoses. Successful telemedicine programs depend on healthcare providers' ability to adopt new technologies effectively [27]. Similarly, Martino et al. [12] highlighted the importance of ongoing training for radiology residents and teleradiologists to improve pediatric trauma imaging accuracy, underscoring the crucial role of technology and training in enhancing pediatric care quality [12].

## Discussion

### Summary of our findings

Our systematic review reveals several key findings regarding the implementation and effectiveness of telemedicine in pediatric emergency surgery and trauma care. First, the synthesis of 11 studies demonstrates consistently high diagnostic accuracy across various applications, with sensitivity ranging from 93.8% to 100% and specificity from 82.6% to 99.7%. These impressive figures suggest that telemedicine can effectively support critical decision-making in pediatric trauma cases. The marked increase in telemedicine consultation requests, particularly exemplified by Zorin et al.'s threefold increase over three years, indicates growing acceptance and integration of this technology into clinical practice [28]. This trend may be attributed to improved technological infrastructure, increased provider familiarity with digital platforms, and the pressing need for specialized pediatric trauma expertise in remote settings [29, 30].

Second, the demonstrated effectiveness in preventing unnecessary transfers (16 cases in Taylor et al.'s study) suggests that telemedicine can optimize resource utilization while maintaining quality of care [25]. This finding is particularly significant given the emotional and financial burden of patient transfers on families and healthcare systems [31]. The comparable performance between radiology residents and attending teleradiologists in trauma CT interpretation (similar discrepancy rates of 13.3%) indicates that telemedicine can effectively support different levels of expertise, potentially expanding the available workforce for pediatric trauma care.

Third, the high satisfaction rates among families and healthcare providers, coupled with improved access to specialized care in rural areas, underscore telemedicine's role in democratizing access to pediatric trauma expertise. The successful implementation of point-of-care ultrasound under remote guidance, achieving 93.8% sensitivity, demonstrates how telemedicine can enhance diagnostic capabilities in resource-limited settings while maintaining quality standards.

These results can be understood within the broader context of healthcare digitalization and the increasing need for flexible, responsive care delivery systems. Worth mentioning is that the COVID-19 pandemic has likely accelerated the adoption and acceptance of telemedicine, creating a more favorable environment for its integration into pediatric emergency care [32, 33].

The successful integration of telemedicine in pediatric trauma care can be explained through the lens of the technology acceptance model (TAM) [34, 35]. Our findings align with TAM's core principles, where perceived usefulness (demonstrated by high diagnostic accuracy) and perceived ease of use (indicated by high provider satisfaction) drive technology adoption. The positive outcomes observed across studies suggest that telemedicine has crossed the critical threshold of provider acceptance, leading to sustained implementation.

### Comparing our results with previous similar systematic review

Our findings largely corroborate and extend previous research in telemedicine applications. Earlier studies focusing on adult emergency care reported similar diagnostic accuracy rates, suggesting that telemedicine's effectiveness translates well to pediatric populations. However, our review reveals unique advantages in the pediatric context, particularly in preventing unnecessary transfers and providing specialized consultation. This differs from adult studies where transfer prevention was less frequently cited as a primary benefit.

The high satisfaction rates among families align with previous research in general pediatric telemedicine, though our review specifically highlights its value in emergency and trauma settings. The successful implementation of remote-guided ultrasound represents an advancement over earlier studies, which primarily focused on store-and-forward imaging applications.

A comparison of our qualitative synthesis with the systematic review by Nguyen et al. [36] reveals key themes that highlight both alignment and divergence in findings [36]. Both reviews emphasize the significant improvement in access to care through telemedicine, particularly for pediatric patients in rural and underserved areas. Our results corroborate the previous SR's findings that telemedicine effectively reduces unnecessary transfers and enhances timely interventions, thereby improving patient outcomes [36].

However, our synthesis expands on the diagnostic accuracy of telemedicine, detailing specific metrics such as sensitivity and specificity for various telemedicine applications, including point-of-care ultrasound and digital imaging. The previous SR also noted high diagnostic reliability but did not delve into the specific metrics as comprehensively as our analysis [36]. Another systematic review by Mitra et al. [14] highlights that telemedicine improves pediatric emergency care by enhancing diagnostic accuracy, reducing length of stay (LOS), improving access to specialized care, and achieving cost savings. However, there is a need for more randomized controlled trials to assess its impact on morbidity and mortality [14].

Furthermore, both reviews acknowledge the importance of training and education for healthcare providers in utilizing telemedicine effectively. While the previous SR highlighted challenges such as technology reliance and potential misdiagnosis [36], our synthesis provides a more detailed exploration of these limitations, emphasizing the need for ongoing training and integration of telemedicine into clinical practice.

### Interpretation and implications

The high diagnostic accuracy across various applications suggests that telemedicine can serve as a reliable tool for initial assessment and ongoing management of pediatric trauma cases. This is particularly relevant for healthcare facilities lacking on-site pediatric surgical expertise. The demonstrated ability to prevent unnecessary transfers while maintaining quality of care implies potential cost savings for healthcare systems and reduced burden on families.

The successful implementation of remote-guided procedures and diagnostics suggests that telemedicine can effectively extend specialized pediatric surgical expertise beyond major centers. This has important implications for addressing healthcare disparities in rural and underserved areas. The comparable performance between residents and attending physicians in teleradiology interpretations suggests opportunities for broadening the workforce while maintaining quality standards.

The evolving role of artificial intelligence (AI) in telemedicine is an exciting future direction. AI can enhance the accuracy and efficiency of telemedicine consultations by analyzing medical images and providing diagnostic support. For example, a study by Martino et al. [12] demonstrated the use of AI-powered computer vision to detect pediatric trauma injuries from CT scans [12]. The study showed that AI can accurately detect injuries and provide diagnostic support to radiologists. The integration of AI in telemedicine has the potential to revolutionize pediatric trauma care by providing faster and more accurate diagnoses.

### Strengths and limitations

Our review benefits from several methodological strengths. The comprehensive search strategy across multiple databases and strict inclusion criteria enhanced the strength of our findings. The inclusion of studies from various countries and healthcare settings improves the generalizability of our results. The systematic quality assessment using validated tools provides confidence in the reliability of our conclusions.

However, several limitations warrant consideration. The relatively small number of included studies (n = 11) and their heterogeneous nature limits the potential for quantitative meta-analysis. The predominance of retrospective studies increases the risk of selection bias. Additionally, the focus on English-language publications may have excluded relevant studies from non-English-speaking regions. The included studies lack detailed information on injury severity, which limits our ability to assess the effectiveness of telemedicine across different levels of trauma. The rapid evolution of technology means that some findings from older studies may not fully reflect current capabilities.

## Summary and recommendations

This systematic review highlights the potential of telemedicine in pediatric emergency surgery and trauma care, demonstrating promising results in diagnostic accuracy, improved access to specialized care, and high satisfaction rates. However, the findings rely on individual studies with varying methodologies, limiting the overall generalizability of conclusions. While the evidence suggests telemedicine can enhance care delivery, further research is needed to establish standardized protocols, assess long-term outcomes, and evaluate cost-effectiveness. Future studies should prioritize prospective designs and economic analyses to better understand the feasibility and sustainability of telemedicine in pediatric trauma care. Integrating telemedicine into existing healthcare frameworks should be pursued with careful consideration of implementation challenges and variability in healthcare settings.

## Supplementary Information

Below is the link to the electronic supplementary material.

Supplementary file1 (DOCX 16 KB)

## Data Availability

The data that support the findings of this study are available upon reasonable request.
