# Supplementary material for: Telemedicine applications in pediatric emergency surgery and trauma: a systematic review of diagnostic accuracy and clinical effectiveness
Source: Pediatr Surg Int. 2025 Apr 22;41(1):122. doi: 10.1007/s00383-025-06023-9 (PMC12014790; doi:10.1007/s00383-025-06023-9)
Supplement: Supplementary file 1 — Supplementary file1 (DOCX 16 KB) [file 383_2025_6023_MOESM1_ESM.docx]

| **Database** | **Search Strategy** |
| --- | --- |
| **PubMed** | ("Virtual Medicine"[Title/Abstract] OR "Medicine, Virtual"[Title/Abstract] OR "Tele-Referral"[Title/Abstract] OR "Tele Referral"[Title/Abstract] OR "Tele-Referrals"[Title/Abstract] OR "Mobile Health"[Title/Abstract] OR "Health, Mobile"[Title/Abstract] OR "mHealth"[Title/Abstract] OR "Telehealth"[Title/Abstract] OR "eHealth"[Title/Abstract] OR "Tele-Intensive Care"[Title/Abstract] OR "Tele Intensive Care"[Title/Abstract] OR "Tele-ICU"[Title/Abstract] OR "Tele ICU"[Title/Abstract] OR "Telecare"[Title/Abstract] OR "Tele-Care"[Title/Abstract] OR "Tele Care"[Title/Abstract] OR telemed*[Title/Abstract] OR tele-med*[Title/Abstract] OR telehealth[Title/Abstract] OR eleconsult*[Title/Abstract] OR teleConsult*[Title/Abstract] OR telepediatric*[Title/Abstract] OR teleradiolog*[Title/Abstract] OR tele-radiolog*[Title/Abstract] OR ehealthcare*[Title/Abstract] OR e-consult*[Title/Abstract] OR "electronic consultation"[Title/Abstract] OR "electronic consultations"[Title/Abstract] OR e-monitoring[Title/Abstract] OR e-assessment[Title/Abstract] OR "digital medicine"[Title/Abstract] OR "digital health services"[Title/Abstract] OR "remote monitoring"[Title/Abstract] OR "wireless health"[Title/Abstract] OR "teleconsultation services"[Title/Abstract] OR "telehealth technology"[Title/Abstract] OR "web-based healthcare"[Title/Abstract] OR "cybermedicine"[Title/Abstract] OR "virtual healthcare"[Title/Abstract] OR "Tele-assistance"[Title/Abstract] OR "Tele-expertise"[Title/Abstract] OR "video conference"[Title/Abstract] OR "Telenursing"[Title/Abstract] OR "Teleneurosurgery"[Title/Abstract] OR "Telerehabilitation"[Title/Abstract] OR "Teletrauma care"[Title/Abstract] OR "Telecardiology"[Title/Abstract] OR "Telesurgery"[Title/Abstract] OR "cybersurgery"[Title/Abstract] OR "Remote surgery"[Title/Abstract] OR "telepresence"[Title/Abstract] OR "online healthcare"[Title/Abstract] OR "internet medicine"[Title/Abstract]) AND (pediatric*[Title/Abstract] OR paediatric*[Title/Abstract] OR child[Title/Abstract] OR children[Title/Abstract] OR adolescent*[Title/Abstract] OR infant*[Title/Abstract] OR neonat*[Title/Abstract] OR newborn[Title/Abstract]) AND (triag*[Title/Abstract] OR emergen*[Title/Abstract] OR trauma[Title/Abstract] OR "urgent operation"[Title/Abstract] OR "crisis surgery"[Title/Abstract] OR "immediate procedure"[Title/Abstract]) |
| **Scopus** | ( TITLE-ABS-KEY ( ( pediatric* OR paediatric* OR child OR children OR adolescent* OR infant* OR neonat* OR newborn ) AND ( triag* OR emergen* OR trauma OR "urgent operation" OR "crisis surgery" OR "immediate procedure" ) ) AND LANGUAGE ( english ) AND ABS ( ( "Virtual Medicine" OR "Medicine, Virtual" OR "Tele-Referral" OR "Tele Referral" OR "Tele-Referrals" OR "Mobile Health" OR "Health, Mobile" OR "mHealth" OR "Telehealth" OR "eHealth" OR "Tele-Intensive Care" OR "Tele Intensive Care" OR "Tele-ICU" OR "Tele ICU" OR "Telecare" OR "Tele-Care" OR "Tele Care" OR telemed* OR tele-med* OR telehealth OR eleconsult* OR teleconsult* OR telepediatric* OR teleradiolog* OR tele-radiolog* OR ehealthcare* OR e-consult* OR "electronic consultation" OR "electronic consultations" OR e-monitoring OR e-assessment OR "digital medicine" OR "digital health services" OR "remote monitoring" OR "wireless health" OR "teleconsultation services" OR "telehealth technology" OR "web-based healthcare" OR "cybermedicine" OR "virtual healthcare" OR "Tele-assistance" OR "Tele-expertise" OR "video conference" OR "Telenursing" OR "Teleneurosurgery" OR "Telerehabilitation" OR "Teletrauma care" OR "Telecardiology" OR "Telesurgery" OR "cybersurgery" OR "Remote surgery" OR "telepresence" OR "online healthcare" OR "internet medicine" ) ) ) |
| **Web of Science (WoS)** | TS=(("Virtual Medicine" OR "Medicine, Virtual" OR "Tele-Referral" OR "Tele Referral" OR "Tele-Referrals" OR "Mobile Health" OR "Health, Mobile" OR "mHealth" OR "Telehealth" OR "eHealth" OR "Tele-Intensive Care" OR "Tele Intensive Care" OR "Tele-ICU" OR "Tele ICU" OR "Telecare" OR "Tele-Care" OR "Tele Care" OR telemed* OR tele-med* OR telehealth OR eleconsult* OR teleConsult* OR telepediatric* OR teleradiolog* OR tele-radiolog* OR ehealthcare* OR e-consult* OR "electronic consultation" OR "electronic consultations" OR e-monitoring OR e-assessment OR "digital medicine" OR "digital health services" OR "remote monitoring" OR "wireless health" OR "teleconsultation services" OR "telehealth technology" OR "web-based healthcare" OR "cybermedicine" OR "virtual healthcare" OR "Tele-assistance" OR "Tele-expertise" OR "video conference" OR "Telenursing" OR "Teleneurosurgery" OR "Telerehabilitation" OR "Teletrauma care" OR "Telecardiology" OR "Telesurgery" OR "cybersurgery" OR "Remote surgery" OR "telepresence" OR "online healthcare" OR "internet medicine") AND (pediatric* OR paediatric* OR child OR children OR adolescent* OR infant* OR neonat* OR newborn) AND (triag* OR emergen* OR trauma OR "urgent operation" OR "crisis surgery" OR "immediate procedure")) |
| **Cochrane Library** | (("Virtual Medicine" OR "Medicine, Virtual" OR "Tele-Referral" OR "Tele Referral" OR "Tele-Referrals" OR "Mobile Health" OR "Health, Mobile" OR "mHealth" OR "Telehealth" OR "eHealth" OR "Tele-Intensive Care" OR "Tele Intensive Care" OR "Tele-ICU" OR "Tele ICU" OR "Telecare" OR "Tele-Care" OR "Tele Care" OR telemed* OR tele-med* OR telehealth OR eleconsult* OR teleConsult* OR telepediatric* OR teleradiolog* OR tele-radiolog* OR ehealthcare* OR e-consult* OR "electronic consultation" OR "electronic consultations" OR e-monitoring OR e-assessment OR "digital medicine" OR "digital health services" OR "remote monitoring" OR "wireless health" OR "teleconsultation services" OR "telehealth technology" OR "web-based healthcare" OR "cybermedicine" OR "virtual healthcare" OR "Tele-assistance" OR "Tele-expertise" OR "video conference" OR "Telenursing" OR "Teleneurosurgery" OR "Telerehabilitation" OR "Teletrauma care" OR "Telecardiology" OR "Telesurgery" OR "cybersurgery" OR "Remote surgery" OR "telepresence" OR "online healthcare" OR "internet medicine") AND (pediatric* OR paediatric* OR child OR children OR adolescent* OR infant* OR neonat* OR newborn) AND (triag* OR emergen* OR trauma OR "urgent operation" OR "crisis surgery" OR "immediate procedure")):ti,ab,kw |

**Supplementary Table 1:** detailed search strategy for each database.
